# Reported use of anti-asthmatic medication in doping control forms from 2015 to 2019 – mapping retrospective data

**DOI:** 10.3389/fspor.2026.1815697

**Published:** 2026-05-15

**Authors:** Aurora Jørnsdatter Kojen, Thomas Halvorsen, Astrid Gjelstad, Stig Larsen, Fredrik Lauritzen, Trine Stensrud

**Affiliations:** 1 Department of Sports Medicine, Norwegian School of Sport Sciences, Oslo, Norway; 2 Department of Clinical Science, Faculty of Medicine, University of Bergen, Bergen, Norway; 3 Science and Medicine, Anti-Doping Norway, Oslo, Norway; 4 Section of Pharmaceutical Chemistry, Department of Pharmacy, University of Oslo, Oslo, Norway; 5 Meddoc Research AS, Skjetten, Norway

**Keywords:** anti-doping, asthma, asthma medication, athletes, sports medicine

## Abstract

**Objectives:**

Asthma and bronchial hyperresponsiveness is common in athletes, particularly in sports with high ventilatory demands or exposure to irritants such as cold, dry air, polluted air, or trichloramines. The mechanisms behind asthma in athletes remain unclear, but repeated high-volume ventilation and exposures to irritants, as well as airway inflammation and remodelling likely contribute. Symptoms resemble those of asthma, and airway hyperresponsiveness plays a key role, even in athletes with no other classic asthma features. Treatment of asthma in athletes follows the same approach as asthma in the general population. Use of asthma medication is commonly reported by athletes on doping control forms (DCFs). Thus, this study aimed to investigate the use of different anti-asthmatic medications among doping-controlled athletes.

**Methods:**

This was an observational cross-sectional study, evaluating retrospective data from athletes′ DCFs obtained during 2015–2019 as part of Anti-doping Norway′s national testing program in Norwegian sport, some of whom reappearing in the data set with multiple forms.

**Results:**

Of 10 418 DCFs, 1702 (16.3%) contained one or more anti-asthmatic medications. Bronchodilators were most frequently reported, with anticholinergics being most common. DCFs reported from females, national level athletes, and athletes with high risk of asthma contained significantly more anti-asthmatic medications compared to their counterparts (*p* *<* 0.01). Usage was highest among athletes aged 30–34, and in those in endurance sports.

**Conclusion:**

Despite the limitation of athletes’ reappearance in the dataset, this study provides insight into guideline adherence and asthma medication use among athletes in Norway. Further research is needed in this area.

## 1 Introduction

Asthma is more prevalent in athletes than in the general population, making it a major medical concern and a leading cause of medication use (1, 2). While exercise generally benefits health, prolonged high-intensity ventilation may negatively affect respiratory health, particularly under certain environmental conditions (3, 4). The etiology and pathophysiology of athletes’ asthma are incompletely understood and likely vary between individuals, but there is a clear association with elite-level endurance training. Sports involving high minute ventilation and exposure to irritants, such as chlorine, polluted air, or cold and dry air, carry higher risk (4–6). Two asthma phenotypes have been proposed: “type 2,” linked to atopic asthma and typically responsive to inhaled corticosteroids, and “non-type 2,” more specific to exercise-induced asthma (5, 7, 8). The parasympathetic nervous system may also be involved, supporting a potential role for anticholinergic treatment (9).

Despite its heterogeneity and complex etiology, athletes’ asthma is generally recommended to be treated according to guidelines for the general population (10, 11). These include self-management strategies and a stepwise medical treatment with increasing doses and additional medications based on symptom severity (11). In athletes, treatment escalation may increase the risk of exceeding doping regulations, as some inhaled *β*2-agonists are permitted only within specified doses, whereas anticholinergics, inhaled corticosteroids, and leukotriene receptor antagonists are allowed. Use beyond permitted limits requires a Therapeutic Use Exemption (TUE) (16, 17).

Poorly managed asthma may limit physical activity not only in athletes but also in the general population, given its high prevalence (12). This may have important public health implications. Understanding how asthma medications are used by athletes is therefore essential, not only at the elite level but also in recreational contexts.

A recent study from Anti-Doping Norway found that anti-asthmatics were the most frequently reported class of medication among athletes, along with painkillers (13); consistent with by earlier literature showing widespread medication use, particularly in endurance athletes (1, 3, 14, 15). This study expands upon Anti-Doping Norway′s previous study by conducting a more detailed analysis of asthma medication use, using the same doping control forms (DCFs) obtained during test sessions conducted in both recreational and elite athletes in Norway, covering the years from 2015 to 2019.

## 2 Methods

This study investigates the use of anti-asthmatic medication based on DCFs obtained during 2015 to 2019 as part of Anti-Doping Norway′s national doping testing program in Norwegian sport, using an observational cross-sectional design, mapping retrospective data. The overall use of pharmaceuticals and dietary supplements from the data set is published elsewhere (13, 16).

The DCFs were obtained between 2015 and 2019 by personnel from Anti-Doping Norway (ADNO), in accordance with the International Standard for Testing and Investigation (ISTI) by WADA (17). Both Norwegian and foreign athletes performing their sport in Norway at events under the auspices of a Norwegian sport federation organized under the Norwegian Confederation of Sport, and Olympic and Paralympic Committee, were tested.

The results of this study are based on DCFs submitted by athletes at the time of testing. In accordance with article 7.4.5 in ISTI, the athlete must report all pharmaceuticals and dietary supplements taken the previous seven days (17). The included DCFs are stored in ADNOs own paper archives in accordance with the World Anti-Doping Code article 14.6 and The Personal Data Act (18, 19).

A dataset for this study was derived from the overall DCF dataset, including the following descriptive variables: sex (male; female), age group (<20; 20–24; 25–29; 30–34; 35–40; >40), athlete level as defined by ADNO—national-level (NLA, competing nationally/internationally) or recreational-level (RLA, below national level)—and sport category (Ball and team; Endurance; Strength and power; Combat; Muscular endurance; Gymnastic; Aiming; Other), categorized by physiological characteristics (20).

A variable was also created to identify athletes in sports considered high risk for asthma, based on Price et al., defined as sports with high ventilation demands and exposure to bronchoconstrictive conditions such as cold, dry, or polluted air, and chlorine products (1). High-risk sports include cross-country skiing, biathlon, swimming, and triathlon; low-risk examples include boxing, soccer, dancing, and climbing.

The dataset organized the pharmaceuticals by their ATC-codes (21) and was for the purpose of this study divided into the following subgroups: Short acting ß2-agonists; Long acting ß2-agonists; Anticholinergics; Leukotriene receptor antagonists; Inhaled corticosteroids. Variables describing the marketed medication were also constructed; Short acting ß2-agonists; Long acting ß2-agonists; Anticholinergics; Inhaled corticosteroids; Combination products; Leukotriene receptor antagonists. In addition, a variable was constructed describing the number of anti-asthmatic medication, by counting the reported number of ATC-codes per DCF.

To ensure a fully anonymized database, all DCFs from 2015 to 2019 were included. As a result, the reappearance of athletes could not be detected, a phenomenon occurring rarely in the RLA group but more frequently in the NLA group, who consists of athletes in the Registered Testing Pool (RTP) or other high-level athletes who are tested several times a year.

Because the overall database is fully anonymized, written consent was not collected from the athletes selected for doping control. As the study is a collaboration between ADNO and the Norwegian School of Sport Sciences, both parts signed a collaboration agreement to secure safe use of the database. The project has been approved by the Regional Committees for Medical and Health Research, ID 29318.

### 2.1 Statistical analysis

Continuously distributed variables are presented as mean values with 95% confidence interval. “Number of doping control forms” within different categories are given in multiple contingency tables. Comparison of groups regarding continuously distributed variable were performed by using Analysis of Variance (ANOVA) with Bonferroni correction. Contingency Table Analysis was used in comparison of “Number of doping control forms” between categories (Ref 2). Differences between groups were classified as significant for *p*-values ≤ 0.05. All analyses were performed using IBM SPSS, version 28.0.

## 3 Results

A total of 10 418 DCFs were included, representing all DCFs collected by Anti-Doping Norway during 2015 through 2019. One or more anti-asthmatic medications were reported in 1702 (16.3%) DCFs (Table 1). Split by substance group as well as by medicine category, bronchodilators were the most frequently reported anti-asthmatic (Tables 2, 3).

**Table 1:** Number of doping control forms reporting anti-asthmatic products, split by sex, athlete level and risk.

Number of products per form	Sex		Athlete level		Risk		Total
	Female	Male	RLA	NLA	Low	High	
0	2023	6693	3902	4814	5436	3280	8716
1	154	532	194	492	185	501	686
2	211	408	97	522	107	512	619
3	62	211	32	241	19	254	273
4	27	51	8	70	1	77	78
5	2	35	1	36	0	37	37
6	0	5	0	5	0	5	5
7	0	4	0	4	0	4	4
Total >0	456	1246	332	1370	312	1390	1702
Total	2479	7939	4234	6184	5748	4670	10418
*P*-values	*p* < 0.01		*p* < 0.01		*p* < 0.01		

RLA, recreational level athlete; NLA, national level athlete.

**Table 2:** Number of doping control forms reporting use of the respective pharmaceutical substance group within age groups, sport category, sex, athlete level and risk.

Factor	Category	Bronchodilators			Anti-inflammatory	
		SABA	LABA	Ach	ICS	LKTR
Age groups	<20	75	28	29	51	12
	20–24	236	188	213	314	50
	25–29	283	218	420	516	54
	30–34	143	104	163	221	19
	35–39	30	32	38	55	8
	>40	31	25	19	32	17
Sport category	Aiming sports	1	2	1	3	0
	Ball and team sports	165	99	16	127	25
	Fighting sports	46	20	14	29	0
	Gymnastics sports	4	1	1	2	0
	Muscular endurance sports	8	2	14	31	0
	Strength and power sports	69	46	75	115	24
	Endurance sports	499	423	761	879	110
	Other	6	2	0	3	1
Sex	Female	151	110	281	327	58
	Male	647	485	601	862	102
Athlete level	RLA	239	130	48	181	33
	NLA	559	465	834	1008	127
Risk	Low	210	131	39	175	22
	High	588	464	843	1014	138
Total		798	595	882	1189	160
		2725			1349	

RLA, recreational level athlete; NLA, national level athlete; SABA, short acting *β*2-agonist; LABA, long acting *β*2-agonist; ICS, inhalation corticosteroids; CP, combination preparate; Ach, anticholinergics; LKTR, leukotriene receptor antagonists.

**Table 3:** Number of doping control forms reporting asthma-related products.

Number of products per form	Bronchodilators			Anti-inflammatory		Combination			
	SABA	LABA	Ach	ICS	LKTR	SABA + ICS*	LABA + ICS*	Ach + ICS*	CP**
RLA 1	127	3	8	10	4	-	-	-	42
2	78	1	16	30	7	23	0	7	60
3	25	5	18	20	14	15	5	13	13
4	8	1	5	5	7	5	1	2	5
5	1	0	1	1	1	1	0	1	1
6	0	0	0	0	0	0	0	0	0
7	0	0	0	0	0	0	0	0	0
Total	239	10	48	66	33	44	6	23	121
NLA 1	85	0	199	72	20	-	-	-	116
2	203	27	313	338	20	83	9	231	135
3	278	38	210	284	31	127	37	167	61
4	52	35	67	62	29	44	33	59	10
5	32	33	36	36	18	32	33	36	3
6	5	5	5	5	5	5	5	5	0
7	4	4	4	4	4	4	4	4	0
Total	559	142	834	701	127	295	121	502	325
	1535			828		1243			
*P*-values total	*p* < 0.01	*p* < 0.01	*p* < 0.01	*p* < 0.01	*p* < 0.01	*p* < 0.01	*p* < 0.01	*p* < 0.01	*p* < 0.01

RLA, recreational level athlete; NLA, national level athlete; SABA, short acting *β*2-agonist; LABA, long acting *β*2 agonist; ICS, Inhalation corticosteroids; CP, combination preparate; Ach, anticholinergics; LKTR, leukotriene receptor antagonists.

* SABA/LABA/Ach + ICS represents forms reporting at least one bronchodilator and one anti-inflammatory product. Ex. Ventoline®/Serevent®/Atrovent® + Flutide®.

** Combination products represent products containing both bronchodilators and anti-inflammatory substances. Ex. Inuxair® or seretide®.

### 3.1 By sex

The number of DCFs in the respective subgroups analyzed are presented in Table 4, with 18.3% of females’ DCFs reporting one or more anti-asthmatic medications, compared to 15.6% of males’ (*p* < 0.01) (Table 1). Among the DCFs reporting one or more anti-asthmatic medications, the use of two medications was more frequently reported among females, while males more frequently reported the use of only one medication. Anticholinergics were the most frequently reported bronchodilator among females, short acting ß2-agonists (SABA) in males. In both sexes, ICS were the most frequently reported anti-inflammatory medication (Table 2).

**Table 4:** Frequencies of doping control forms included in the study, split by categories.

Factor	Category	Age distribution						Total
		<20	20–24	25–29	30–34	35–39	≥40	
Sex	Female	376	805	883	214	100	101	2479
	Male	1086	2814	2476	1051	267	245	7939
Athlete level	RLA	958	1419	1053	386	174	244	4234
	NLA	504	2200	2306	879	193	102	6184
Sport Category	Aiming sports	27	24	20	8	5	22	106
	Ball and team sports	689	1569	1246	429	103	21	4057
	Fighting sports	200	349	140	70	26	14	799
	Gymnastics sports	38	55	32	3	1	1	130
	Muscular endurance sports	7	53	80	37	7	0	184
	Strength and power sports	203	548	616	219	94	78	1758
	Endurance sports	272	990	1187	482	112	190	3233
	Other	26	31	38	17	19	20	151
Risk	Low	1059	2096	1637	625	202	129	5748
	High	403	1523	1722	640	165	217	4670
Total		1462	367	3619	3359	1265	346	10418

RLA, recreational level athlete; NLA, national level athlete.

### 3.2 By age group

**Figure 1:**
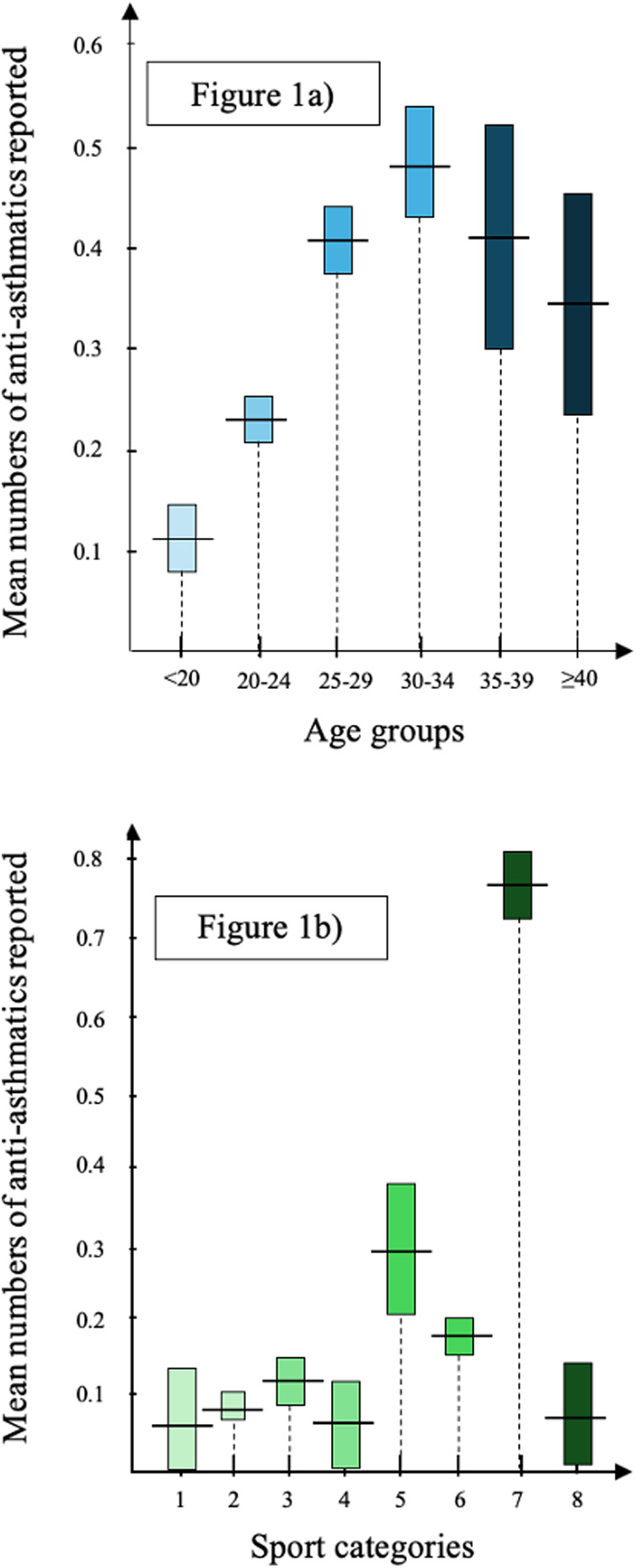
Mean numbers of anti-asthmatic products. **1a**) divided in age groups and **1b**) in sport categories. The results are expressed as mean values illustrated by the horizontal line crossing the 95% Confidence Interval columns 1 = Aiming sports; 2 = Ball and team sports; 3 = Combat sports; 4 = Gymnastics sports; 5 = Muscular endurance sports; 6 = Strength and power sports; 7 = Endurance sports; 8 = Other sports.

The age groups with the highest reported medication use were 25–29 years (34.7%) and 30–34 years (32.2%). The number of reported medications increased with age, peaking at a mean of 0.48 medications per DCF in the 30–34 age group before declining (Figure 1a, all *p* < 0.01). SABA was the most common bronchodilator in the <20, 20–24, and >40 age groups, while anticholinergics were most reported in the 25–39 age range. SABA was the least reported bronchodilator, except in the <20 and >40 age groups, where long acting ß2-agonists (LABA) and anticholinergics, respectively, were least used (Table 2).

### 3.3 By athlete level

RLA accounted for 40.6% of DCFs, while NLA accounted for 59.4% (Table 4). NLA DCFs contained significantly more anti-asthmatic medications, with 22.2% reporting one or more, compared to 7.8% for RLA (*p* < 0.01). NLA most frequently reported two medications, while RLA reported one (Table 1). By substance group, anticholinergics were the most common bronchodilator among NLA, while SABA was most frequent in RLA. ICS was the most reported anti-inflammatory for both groups (Table 2). NLA DCFs contained significantly more anti-asthmatic medications (*p* < 0.01), except for single SABA medications, which were more common in RLA (*p* < 0.01) (Table 3). RLA most frequently reported combination medication (n = 121), while NLA most often reported ICS and anticholinergics together. The highest number of medications was found in NLA DCFs, with four cases reporting seven medications each.

### 3.4 By sport category

Ball and team sports (38.9%) and endurance sports (31%) were the most common sport categories on DCFs (Table 4). Endurance sports had the highest mean number of reported medications (0.77, *p* < 0.01), followed by muscular endurance sports, which reported significantly more medications than aiming, ball and team, combat, gymnastics, and other sports (*p* < 0.01) (Figure 1b).

Endurance athletes reported significantly higher use of all medications compared to other sports (all *p* < 0.01) (Table 2). ICS was the most frequently reported anti-inflammatory medication across all sports, with the highest prevalence in endurance athletes. SABA was the most reported bronchodilator in ball and team, combat, gymnastics, and other sports, while anticholinergics were most common in endurance, strength and power, and muscular endurance sports. LABA was the most reported bronchodilator only in aiming sports (Table 2).

### 3.5 By risk for asthma

Of the 10,418 DCFs, 44.8% were from athletes at high risk of developing asthma (Table 4). DCFs from high-risk sports reported significantly more anti-asthmatic medications than those from low-risk sports (*p* < 0.01) (Table 1). Among high-risk sports, 29.7% of DCFs reported one or more medications, compared to 5.4% for low-risk sports. High-risk athletes most often reported using two medications, while low-risk athletes reported one. Anticholinergics were the most common bronchodilator among high-risk athletes, and SABA among low-risk athletes. ICS were the most frequently reported anti-inflammatory medicine in both groups (Table 2).

## 4 Discussion

### 4.1 Key results

This study analyzed 10,418 DCFs obtained during 2015 to 2019 as part of Anti-Doping Norway′s national doping testing program in Norwegian sport, providing a unique overview of asthma medication use in sport. Approximately one in six DCFs reported anti-asthmatic medications, with use being most prevalent among NLA and endurance athletes. Inhaled corticosteroids were the most frequently reported anti-inflammatory agents, while anticholinergics dominated among bronchodilators. Patterns also differed by athlete level, with short-acting *β*2-agonists most commonly reported by RLA and anticholinergics by NLA.

Research on anti-asthmatic medication use in athletes is scarce. Fitch examined the use of *β*2-agonists among Olympic athletes and found TUE approval rates increasing from of 4.2% to 7.8% between 2002 and 2010 (14, 15). Alaranta et al. reported 7% medication use among 446 elite Finnish athletes (22). Locke and Marks found that 9% of 424 elite New Zealand athletes using anti-asthmatics (23). The higher reported usage of 16% in our study may be due to repeated appearances of the same athletes across different DCFs in the NLA group.

Gjelstad et al., found anti-asthmatics to be the most commonly reported pharmaceuticals in the DCFs, consistent with the high prevalence rates we see when analyzing the same dataset (13), reflecting asthma as a common health issue among athletes (4, 8). A recent study estimated an overall asthma prevalence of 21.8% among athletes since 1990, with a higher rate of 25.1% in endurance athletes (1). Compared to other studies on medication use, our results align more closely with these prevalence rates, which seems reasonable as affected athletes would be expected to use asthma medication (3, 14, 15, 22).

Females reported higher use of asthma medication than males (18.3% vs. 15.6%), consistent with the overall pattern found by Gjelstad et al., reporting greater pharmaceutical use among females (13). This aligns with the asthma prevalence rates, which is generally higher in female athletes (1). Few studies have compared use of anti-asthmatic medication between sexes, but Locke and Marks found significantly higher usage among females, reflecting our findings (23). However, their reported prevalence was lower, possibly due to differences in sample size and athlete reappearance in DCFs in the present study.

The age distribution showing highest usage in the early 30s may be explained by the higher risk of developing asthma after many years of excessive high-intensive training (24–27). This observation aligns with findings by Locke and Marks, who similarly noted a heightened prevalence of medicine use among older athletes relative to their younger counterparts (23). However, the age groups included in this study are older compared to the study from Locke and Marks where the oldest age group was 25–30 years.

NLA reported a higher use of anti-asthmatic medication compared with RLA, again reflecting the pattern for overall pharmaceutical use described by Gjelstad and colleagues, with a higher reported use reported in DCFs from NLA compared with RLA (13). To our knowledge, no other studies have compared the use of anti-asthmatic medications between NLA and RLA, and there are no studies mapping the use of anti-asthmatics in RLA alone.

Interestingly, DCFs from RLA showed a higher frequency of single-use SABA medicine than those from NLA (Table 3). Since 2019, the GINA guidelines have discouraged SABA monotherapy in asthma treatment due to potential negative health outcomes when ICS is not included (11, 28). This change is based on the understanding that asthma is primarily an inflammatory condition. As the data in this study were collected prior to this update of the GINA guideline, the new recommendations may have led to shifts in clinical practice and usage patterns. Future research should revisit this issue considering the updated guidelines.

The most reported bronchodilator was anticholinergics (Tables 2, 3), consistent with Bernhardsen et al. (3). Among cross-country skiers, anticholinergics were the most used bronchodilator (62.5%), followed by SABA (43.8%) and LABA (40.6%) (3). However, *β*2-agonists were more common than anticholinergics among rowers and swimmers. These discrepancies may be influenced by athlete reappearance in our study, as the high prevalence of anticholinergics was particularly notable in NLA DCFs, where reappearance was likely most frequent.

Several factors may explain the high prevalence of anticholinergics in NLA DCFs. Some studies have shown that increased parasympathetic activity is associated with BHR in athletes (9), leading to acetylcholine-induced bronchoconstriction. Anticholinergics block muscarinic receptors, preventing this process and potentially offering better symptom relief than *β*2-agonists, particularly for highly committed athletes like NLAs (5). Since DCFs only document medication use, medical diagnoses remain uncertain. Another possible explanation is that anticholinergics are not on WADA's prohibited list, unlike *β*2-agonists (29). To our knowledge, no studies have examined anticholinergics potential performance-enhancing effects. Further research is needed to assess their efficacy as bronchodilators and ensure both health and fair play.

Endurance athletes had the highest mean number of reported medications, reflecting their greater asthma prevalence compared to athletes in other sports (1). Like older age groups, this may result from years of high-intensity training. Many DCFs in this category come from athletes in sports exposed to bronchial irritants, such as cold, dry, and polluted air, or trichloramines from swimming pools, which increase the risk of developing asthma (4). This also explains why muscular endurance sports, like speed skating and alpine skiing, had the second highest mean number of reported medications, due to frequent exposure to such irritants.

Split by the potential risk for asthma, 29.7% of the DCFs from athletes in high-risk sports contained one or more anti-asthmatic medications, compared to 5.4% among athletes from low-risk sports. This reflects the prevalence of asthma among high-risk and low-risk sports, where sports with high ventilation demands and exposure for bronchial triggers are of high risk to develop asthma. Bernhardsen et al., found significantly higher use of anti-asthmatic medications in typically high-risk sports than in ball and team sports, consistent with our findings (3).

This study provides valuable insight into the prevalence and types of asthma medications used among athletes, contributing to a better understanding of how such medications are utilized within a sporting context. Future studies should investigate dosing patterns and usage in greater detail, including the prevalence of prohibited substance use, the extent of TUE, and the number of adverse analytical findings and anti-doping rule violations associated with asthma medications.

### 4.2 Limitations

While the study benefits from a substantial sample size, a key limitation is that DCFs do not directly correspond to individual athletes, so the same athlete may have submitted multiple forms, potentially skewing the results. This is expected to be more pronounced in the NLA group, particularly among RTP athletes, who must report whereabouts and undergo multiple annual tests (30). Registered testing pool athletes compete in sports with higher doping risk, requiring frequent out-of-competition testing, for example in endurance sports. Consequently, generalizability is likely reduced in this group compared with RLA athletes, who are less frequently tested.

As the database is fully anonymized, the results are based on self-reported usage of anti-asthmatic medication, thereby enabling reporting bias as a possible limitation. As mentioned by Gjelstad et al., the doping control process can induce stress in athletes, potentially leading to forgetfulness and underreporting (13). Simultaneously, it is worth considering whether some athletes may overreport out of an abundance of caution. If necessary, athletes do have the option to submit post-registration to ADNO. However, neither data pertaining to post-registration nor urine test results were included in this study due to its complete anonymization.

During the study period (2015–2019), only inhaled salbutamol, formoterol, and salmeterol were permitted under the WADA regulations, and only within specified dose limits; all other *β*2-agonists were prohibited unless covered by a TUE. However, we did not have access to information on TUE status, nor sufficiently detailed data on dosage and administration to assess compliance with these regulations. As such, we were unable to determine whether the reported use constituted permitted or prohibited use. Therefore, this study does not address potential anti-doping rule violations but rather focuses on describing the prevalence and patterns of asthma medication use among athletes.

## 5 Conclusion

This study analyzed 10,418 DCFs and found that 16.3% reported the use of one or more anti-asthmatic medications. NLA and endurance athletes had the highest usage rates, with anticholinergics being the most frequently reported bronchodilator. A key limitation is that the dataset does not correspond to individual athletes, meaning some may appear multiple times, potentially skewing the results. Nevertheless, the findings align with known prevalence rates of asthma in athletes, supporting the validity of the data. Future research should investigate individual usage patterns and the role of anticholinergics in both health and performance contexts.

## Data Availability

The data analyzed in this study is subject to the following licenses/restrictions: The raw data supporting the conclusions of this article are not publicly available due to ethical and privacy considerations. Requests to access these datasets should be directed to fredrik.lauritzen@antidoping.no.
